# In-depth health surveillance and clinical nutrition in farmed Atlantic salmon: a strategic attempt to detect and mitigate an HSMI outbreak

**DOI:** 10.1186/s13567-023-01137-1

**Published:** 2023-01-24

**Authors:** Johan Rennemo, Steinar Myrvold, Kjetil Berge, Øyvind Kileng, Børge Pedersen, Dan Sindre Aksberg, Piotr Lisik, Delphine Crappe, Charles McGurk, Espen Rimstad, Øystein Wessel, Erling Olaf Koppang, Håvard Bjørgen

**Affiliations:** 1 Skretting AS, Stavanger, Norway; 2 Ellingsen SeaFood AS, Lofoten, Norway; 3 Skretting Aquaculture Innovation (AI), Stavanger, Norway; 4 Unit of Virology, Faculty of Veterinary Medicine, Norwegian University of Life Sciences, 1433 Ås, Norway; 5 Unit of Anatomy, Faculty of Veterinary Medicine, Norwegian University of Life Sciences, 1433 Ås, Norway

**Keywords:** Blood analysis, clinical nutrition, fish welfare, fish health, health monitoring, histopathology, HSMI, population medicine, PRV-1

## Abstract

Fish health personnel have limited tools in combatting viral diseases such as heart and skeletal muscle inflammation (HSMI) in open net-pen farmed Atlantic salmon. In this study, we aimed to predict HSMI by intensified health monitoring and apply clinical nutrition to mitigate the condition. We followed a commercial cohort (G1) of Atlantic salmon that was PRV-1 naïve when transferred to a sea cage at a location where HSMI outbreaks commonly occur. The fish in the other cages (G2-G6) at the location had a different origin than G1 and were PRV-1 positive prior to sea transfer. By continuous analysis of production data and sequentially (approximately every fourth week) performing autopsy, RT-qPCR (for PRV-1 and selected immune genes), blood and histological analysis of 10 fish from G1 and G2, we identified the time of PRV-1 infection in G1 and predicted the onset of HSMI prior to any clinical signs of disease. Identical sequences across partial genomes of PRV-1 isolates from G1 and G2 suggest the likely transfer from infected cages to G1. The isolates were grouped into a genogroup known to be of high virulence. A commercial health diet was applied during the HSMI outbreak, and the fish had low mortality and an unaffected appetite. In conclusion, we show that fish health and welfare can benefit from in-depth health monitoring. We also discuss the potential health value of clinical nutrition as a mean to mitigate HSMI.

**Supplementary Information:**

The online version contains supplementary material available at 10.1186/s13567-023-01137-1.

## Introduction

Open-net pen farmed Atlantic salmon (*Salmo salar*) are exposed to a variety of bacterial, viral, and parasitic agents [1]. Although efficient vaccines targeting bacterial diseases were implemented in the 1990s [2, 3], commercial vaccines against viral diseases have been less successful or even lacking, as is the case with heart and muscle skeletal inflammation (HSMI) caused by infection with Piscine orthoreovirus 1 (PRV-1) [4, 5]. Furthermore, the repertoire of clinical tools for diagnostic or treatment purposes for field-working veterinarians is limited and novel therapies and improved fish health strategies are constantly sought.

PRV-1 has been detected in all countries with large scale farming of Atlantic salmon, including Norway, Canada, Scotland, and Chile. HSMI appear to be prevalent in all these countries, except Canada where only a limited number of non-clinical HSMI cases has been recorded. In Norway, HSMI was first reported at the end of the 1990s as an emerging disease. The disease was first observed in Mid-Norway and soon found on farms all along the Norwegian coast [6]. A viral cause was suspected, and a virus was detected in 2010 [4], but the causal relationship with PRV-1 was not demonstrated until 2017 [5]. HSMI was excluded from the list of notifiable diseases of the national Fish Health Authorities in 2014 due to its highly prevalent occurrence and widespread distribution of its causative agent PRV-1.

HSMI diagnosis is suspected upon the observation of clinical signs of disease at autopsy, often seen as circulatory failure with ascites, pericarditis and swollen liver, and confirmed when PRV-1 is detected by RT-qPCR (varying loads) accompanied by histopathological changes in the heart ventricle and in the red skeletal muscle [7]. Additionally, commonly occurring conditions like cardiomyopathy syndrome (CMS) and pancreas disease (PD) need to be ruled out. The accumulated mortality in an HSMI outbreak varies from insignificant up to 20% [7]. The rate of infection, however, will usually reach 100%, but the time of infection can differ substantially between fish groups: some are infected in the hatchery or as early as pre-smolt or smolt, while others are infected during the seawater phase [8, 9]. Nevertheless, not all PRV-1 infected fish develop HSMI. The outcome of either early or late infection is not fully understood, as other factors such as the general health status of the fish, environmental conditions, and differences in PRV-1 isolates involved can influence HSMI development. Virulence differences between PRV-1 isolates were recently demonstrated using a standardized dose of virus isolates in a challenge experiment where all non-viral parameters were as similar as possible between the groups. Isolates differed in their ability to induce the HSMI heart lesions and sequence analysis indicated that this property was linked to the five genomic segments (L1, L2, M2, S1, S4) of PRV-1 [10]. Thus, the virus isolates can be categorized into genogroups based on the combination of these five genomic sequences. A survey of PRV-1 isolates from salmon farms in Norway demonstrated the presence of viruses belonging to genogroups of both high and low virulence [11]. Further investigations where targeted mapping of PRV-1 isolates from fish suffering HSMI would increase our understanding of the pathogenesis of this highly prevalent disease.

Clinical nutrition is vital in treatment of several diseases in companion animals [12]. For production animals, such diets are less common, but they are receiving increasingly more attention for farmed salmon. This is particularly true for diseases affecting the circulatory system, such as HSMI. Several studies have shown that the impact of HSMI can be modulated by clinical nutrition and functional feeds [13, 14]. However, health diets represent an extra cost for the farmer, and an obvious challenge for the field-working veterinarian is knowing when to apply such feeds to ensure the best possible health effect versus costs. A systematic approach to address this question could be done by large-scale testing in a relevant production setting.

In this study, both PRV-1 negative (G1) and PRV-1 positive smolt (G2-G6) were transferred from fresh water to their respective sea cages at a field location where HSMI historically repeatedly had occurred. The fish in G1 and G2 were closely monitored throughout the first 12 months at sea. Continuous analysis of production parameters and frequent investigations of the gene expression and PRV-1 infection status, histological examination, and blood analysis pinpointed the onset of HSMI and enabled application of clinical nutrition to potentially counteract the impact of disease. We discuss the relevance of the multimodal approach, the use of clinical nutrition in relation to HSMI as well as practical considerations of the handling of PRV-1 infection in farmed salmon.

## Materials and methods

### Fish stock and sampling

**Figure 1:**
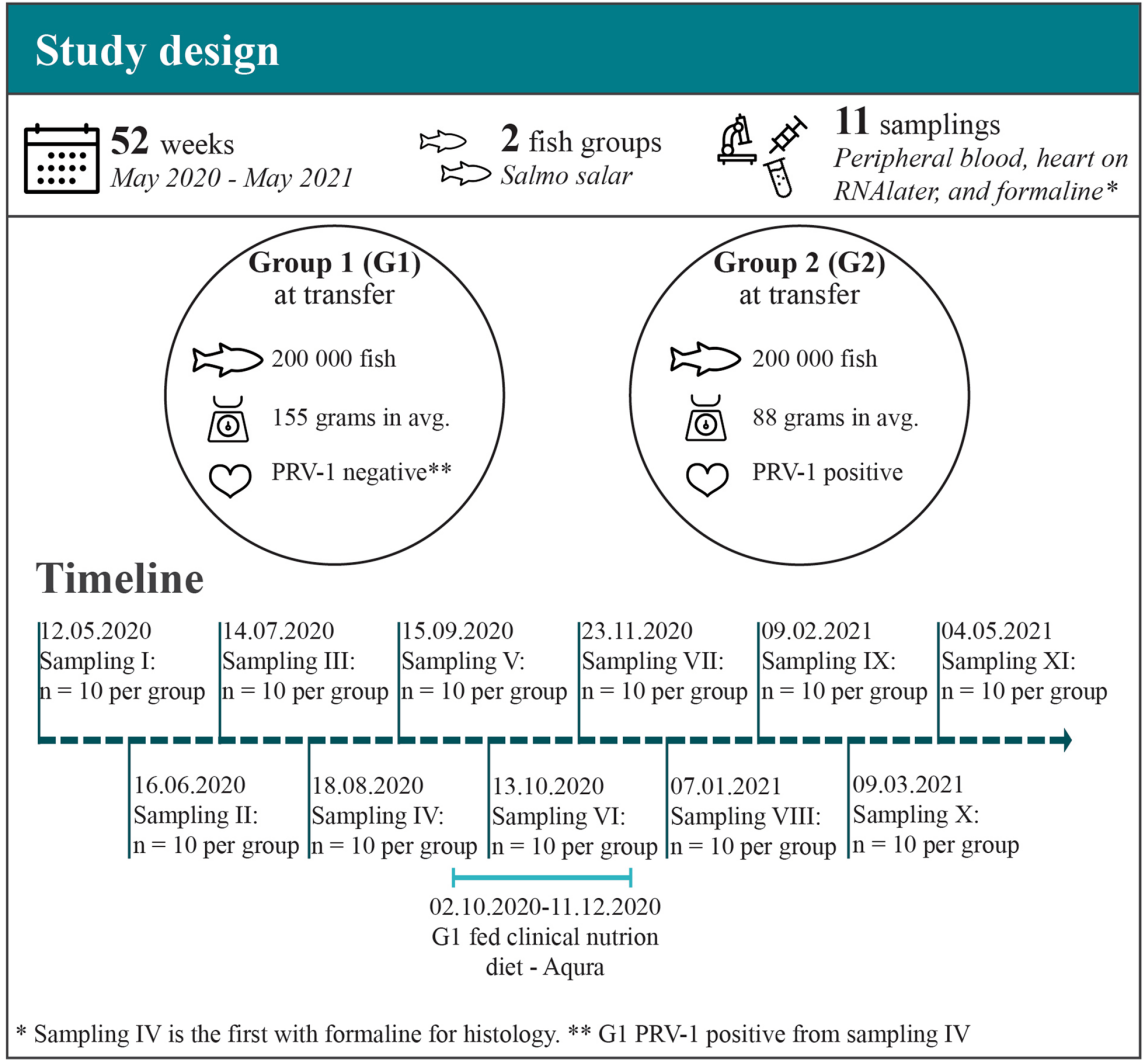
**Study design including key events and samplings.**

Approximately 200 000 Atlantic salmon smolts of the AquaGen SHIELD strain with average weight of 155 g and PRV-1 negative by RT-qPCR (*n* = 10), were transferred to sea cage 1 (G1) at Ellingsen Seafood’s location Stabben in Ofotfjorden (Northern Norway) in May 2020. Approximately 1 000 000 Atlantic salmon smolts of the AquaGen GAIN red strain with average weight of 88 g and PRV-1 positive by RT-qPCR (*n* = 10), were transferred to the same location and distributed in sea cages 2–6 (G2-G6) in May/June 2020 (Figure 1). The cages were organized in one line with a distance of 45 m between each cage. All fish had been vaccinated with Alpha Ject micro 6 (Pharmaq part of Zoetis) in due time prior to sea transfer (G1 in December 2019 and G2 in February 2020). The vaccine stimulates protective immune responses towards bacteria causing furunculosis, coldwater vibriosis, classic vibriosis, winter sores and virus causing infectious pancreas necrosis.

The coming year after sea transfer, i.e. from May 2020 to May 2021, eleven (I-XI) samplings were conducted at approximately 4 week intervals. To ensure a representative sampling of fish, a large net was lowered into the cages (G1 and G2) during feeding and was rapidly pulled through the water. This procedure ensured sampling of fish engaged in feeding. Only fish found representative of the population within the sea cage were further investigated, i.e. individual fish deemed as outliers due to wounds, affected gills, low growth etc. were excluded.

The samplings were performed in accordance with national regulations for animal welfare (Forskrift om drift av akvakulturanlegg § 34. Avlivning av fisk). At each sampling, 10 fish from G1 and 10 fish from G2 were euthanized by a lethal dose of anesthetics (Benzoak vet., ACD Parmaceuticals AS). Following euthanasia, peripheral blood (in heparin) was collected. The fish were autopsied, and heart samples were collected in RNAlater for downstream viral- and immune gene expression analyses by RT-qPCR (performed by PatoGen Analyse, Ålesund, Norway and Skretting ARC, Stavanger, Norway, respectively). At the last eight samplings from August 2020, heart- and side-line samples including skin, red- and white muscle were collected in 10% phosphate buffered formalin for histological analysis performed at NMBU, Faculty of Veterinary Medicine, Ås, Norway.

### Analysis of production data

Production data, i.e., appetite- and mortality numbers, were monitored daily in G1 and G2. Appetite was registered, while mortality numbers were accounted for by daily registrations from fish farm personnel.

### RT-qPCR

#### RNA isolation

Total RNA was isolated from heart samples using the RNeasy Fibrous Mini Kit (QIAGEN, Hilden, Germany). Heart samples were removed from RNAlater and transferred to Buffer RLT with 1 mM DTT. Homogenization of heart tissue was performed with the use of TissueLyserII (QIAGEN) and 5 mm stainless steel beads. Tissue lysis was performed for 5 min with 25 Hz. After lysis, samples were centrifuged for 5 min at >12 000 rct to collect tissue debris. RNA isolation was performed following the manufacturer’s protocol. RNA was eluted with RNAse free water and stored at −80 °C.

#### Reverse transcriptase polymerase chain reaction

Synthesis of first strand cDNA was performed using High Capacity cDNA Reverse Transcription Kit (Thermo Fisher Scientific, Waltham, USA), random/oligoDT primers. A total of 2 µg of RNA was used as a template for RT-PCR reaction. Heart cDNA was later used as a template for qPCR experiments.

#### RT-qPCR

Quantitative PCR was run in QuantStudio 5 (Thermo Fisher Scientfic) and SYBR-Green was used for detection. The specificity of the SYBR Green assays were confirmed by melting point analysis. RT-qPCR reaction consisted of 10 µL PowerUP SYBR Green Mix (Thermo Fisher Scientific), forward and reverse primers, and 1 µL of cDNA diluted 1:10 with nuclease-free water. Standard cycling conditions and primer concentrations were optimized. Average expression was calculated based on two technical replicates that were normalized against the average reference genes: *eukaryotic translation initiation factor 3* (eIF3) and *b-actin*. The relative amount of mRNA of the gene of interest was expressed as a fold change relative to the reference genes (ΔCt = Ct_gene of interest_ – Ct_average of housekeeping genes_) and was calculated for the individual fish. Median ΔCt was calculated for 10 individuals for every sampling point and used on graphs. The selected genes were *Mx1-2*, *GIG2* and *Viperin*, related to early immune/antiviral responses and CD8b and GZMa related to T cell immune responses. *Mx*, *GIG-2* and *Viperin* were investigated due to their function as interferon-induced genes with antiviral properties (VRG – virus responsive genes), especially RNA viruses [15]. CD8b and GZMa were investigated due to the known importance in the adaptive immune response towards PRV-1 infection [16, 17]. See Additional file 1 for primer details.

### Statistical analysis

**Figure 2:**
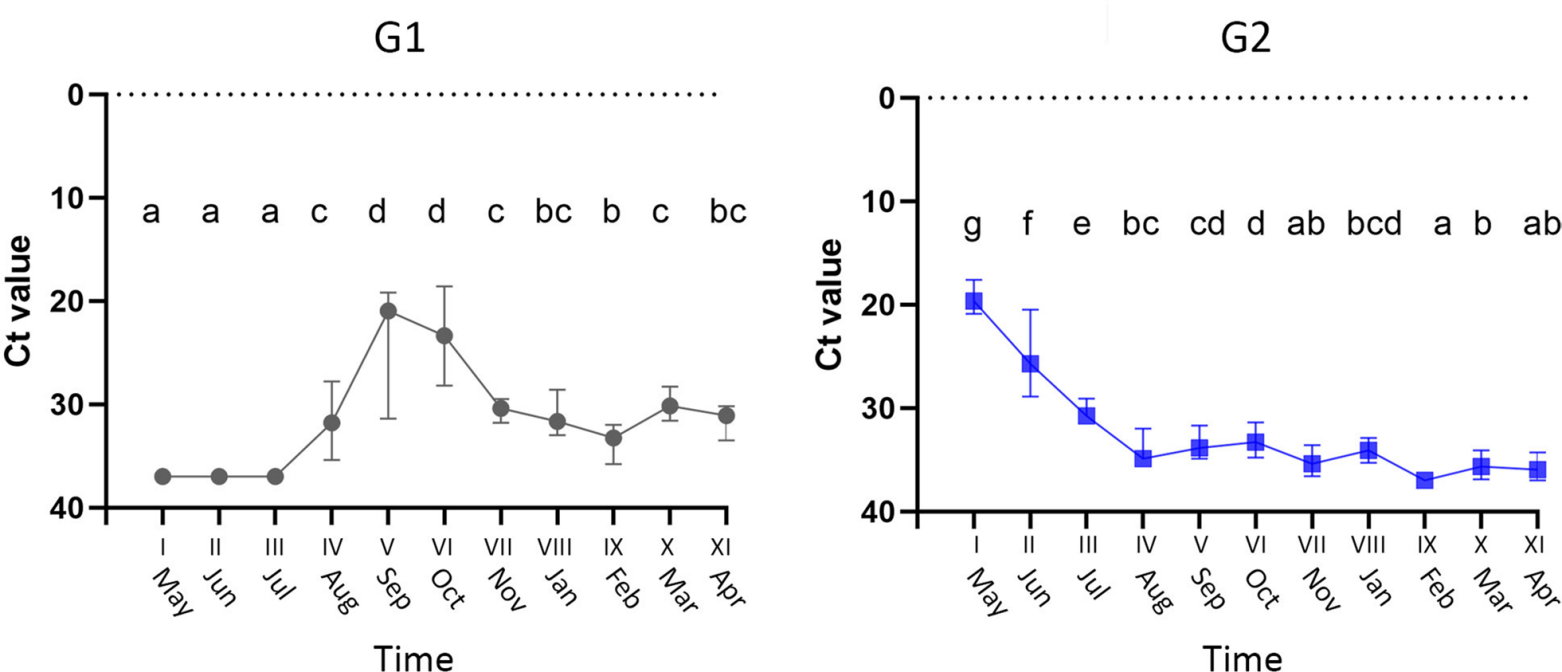
**PRV-1 RNA levels in G1 and G2 measured by reverse transcription-quantitative polymerase chain reaction (RT-qPCR) at each sampling time point (I-XI**, ***n*** **= 10).** Data presented as median Ct values. Time points not connected by the same letter are significantly different (Student’s *t* test).

**Figure 3:**
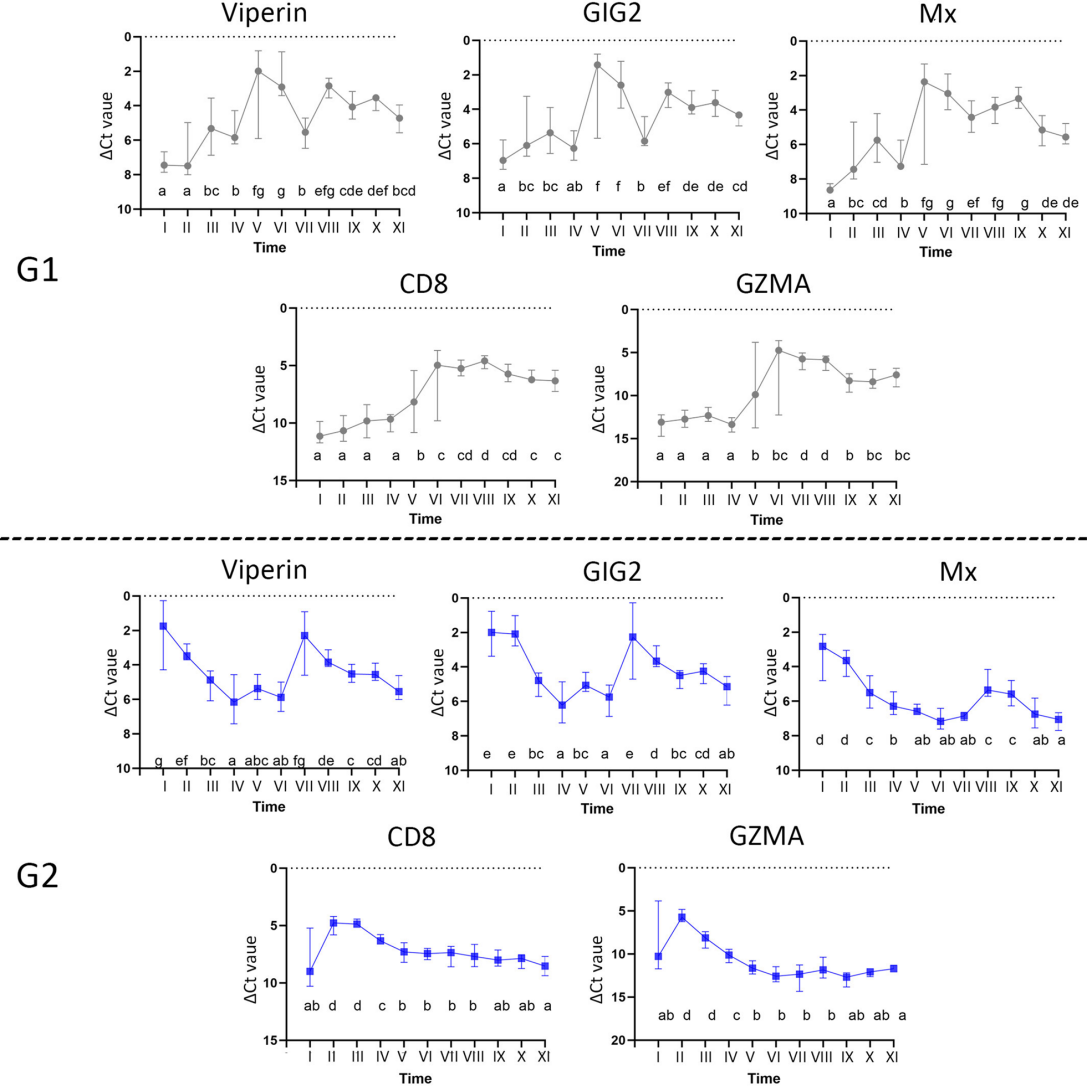
**Expression of Mx, GIG2, Viperin, CD8 and GZMA in heart samples from 10 fish at each time point in G1 (upper) and G2 (lower).** The relative expression (median values) is visualized as ΔCt values indicated on the y axis, while different sampling points are indicated on the x axis. Time points not connected by the same letter are significantly different (Student’s *t* test).

RT-qPCR of PRV-1 and immune genes were analyzed. Means at the different time points were compared using the Student’s *t* test. A connecting letters report indicates differences between the time points. Levels not connected by the same letter are significantly different. Letters have been added to Figures 2 and 3.

### Histological investigations

Samplings IV-XI (from August 2020 until May 2021) included heart and side-line samples with both red and white skeletal muscle for histological investigations. The formalin-fixed samples were dehydrated through graded ethanol baths, cleared in xylene, and embedded in paraffin. Sections of 2 μm were made from all collected samples and were subjected to rehydration and stained with hematoxylin and eosin (HE) according to standard protocol.

The sections were evaluated for degeneration, necrosis, inflammation, and regeneration. In heart samples, the atrium, ventricle and bulbus were investigated and in muscle samples, both red and white muscle were evaluated. The sections were investigated at 200X and scored for HSMI-related lesions using a scale from 0 to 3, where 0 was no changes, 1 mild changes, 2 moderate changes and 3 severe changes. To obtain the mild score (1), only focal changes were observed, while moderate scores (2) were characterized by multifocal changes and severe scores (3) had confluent and diffuse changes.

### Blood analysis

Blood samples were taken from tail vessels (*v./a. caudalis*) by Vacuette containers containing lithium-heparin. The blood samples were pooled and centrifuged at the farming site immediately after sampling (10 min, 3000 *g*, room temperature) and plasma was transferred to Eppendorf tubes and frozen at −80 °C until analysis for the presence of plasma enzymes creatine kinase (CK) and aspartate transaminase (AST). CK is known to increase in plasma after muscle damage and has been shown to correlate with increased histopathological changes in fish with HSMI [18]. AST is known to increase in plasma after muscle and/or liver damage [19]. The parameters were analyzed using a Konelab 30i (Thermo Fisher Scientific).

### PRV-1 sequencing and sequence analysis

Two samples were selected for PRV-1 sequencing, one from each group/cage. From G2 (that was PRV positive prior to sea transfer), one fish with Ct 16.9 was selected. From Group 1, one fish with confirmed HSMI, Ct 25.7 and with histological heart score 3 and red muscle score 3, was selected. Partial genome sequencing was performed targeting the five PRV-1 segments L1, L2, M2, S1 and S4 linked to virulence. The sequencing was performed by PatoGen AS (Ålesund, Norway) as previously described [11]. Multiple sequence alignments were performed using AlignX (Vector NTI Advance 11.0) and phylogenetic analysis was performed using Mega X v.10.17) as previously described [11].

### Health surveillance

After each sampling, results from production data, autopsy, RT-qPCR, blood analysis and histological investigations were obtained within 1 week. Trends (increasing, decreasing or steady values) in the population from one sampling to the next were evaluated for the different methods. The results were discussed in the project group with regards to HSMI development and when to initiate feeding with *Aqura*^*tm*^ - a clinical nutrition diet owned and commercialized by Skretting AS (Stavanger, Norway).

### Nutrition

The fish in G1-G6 was fed a standard grower diet in the seawater phase. When the onset of HSMI was suspected in G1, the diet was changed to *Aqura*. *Aqura* was fed for 10 weeks in G1 (2. October – 11. December) in accordance with the producers’ guidelines. In brief, *Aqura* differs from standard grower diets by several criteria: lower lipid content, higher protein content and a higher marine content. The minimum level of EPA + DHA was 14% of total fat in the feed. In addition, *Aqura* contains elevated levels of several vitamins, minerals, and other functional ingredients.

## Results

### PRV-1 detection (RT-qPCR)

Prior to sea transfer, the fish in G1 was found PRV-1 negative (Ct ≥ 37). Concurrently, representative fish designated for G2 were confirmed PRV-1 positive (median Ct value 19.65). After sea transfer, median PRV-1 Ct value in G2 increased to 25.7 (June) and 30.75 (July). In the remaining samplings, the Ct values were consistently high (>33) or not detected in G2. Note that PRV-1 was not detected in G2 in sampling IX.

The fish in G1 was PRV-1 negative until sampling IV (August). In sampling III, the fish sampled (*n* = 10) in G1 were all found PRV-1 negative, while at sampling IV all fish were found PRV-1 positive. Lowest median Ct value was 20.95 in sampling V (September). From sampling V, the median Ct value steadily increased to sampling IX and remained > 30 throughout the observation period (Figure 2).

### PRV-1 sequencing identified high virulent isolate

The PRV-1 nucleotide sequences obtained from group G1 and G2 were identical, indicating that virus from G2 was transmitted to G1. The phylogenetic analysis of the five genomic segments (L1, L2, M2, S1, S4) showed that the nucleotide sequence of the isolate in the present study was identical to that of a previously identified isolate called NOR2019 NRL-1742 m (GenBank accession number MW831760-MW831764) [11]. This isolate clustered into genogroup “High-2”, which is of high virulence as defined by reference isolate NOR2018-SF [5].

### Immune gene expression analysis

In G1, the ΔCt value of the expression of early anti-viral response genes: *mx, gig2* and *viperin* showed increased expression in sampling V (September) and sampling VI (October). The expression pattern varied in the remaining samplings (VII-XI) but were lower than in sampling V and VI (Figure 3A), i.e. the expression of the early anti-viral response genes followed the load of PRV-1 RNA as seen by RT-qPCR (Figure 2).

In G2, the ΔCt value of the expression of *mx, gig2* and *viperin* in sampling I (May) was at the same level as G1 was at sampling V, i.e. at peak expression. The early anti-viral response genes in G2 were therefore assessed as high in samplings I (May) and II (June), followed by down-regulation of these genes. *Gig2* and *Viperin* expression was also increased in sampling VII (Figure 3A).

Analysis of the cytotoxic T-cell response in G1 revealed increased expression of *cd8b* and *gzma* in samplings VI (Figure 3B), i.e. delayed by one sampling period relative to the increased expression of early anti-viral response genes (Figure 3A). The expression level remained higher than in samplings I-V in G1 throughout the study. In G2, the highest expression was registered in sampling II, again delayed by one sampling period relative to early anti-viral response genes, and the expression pattern showed a decreasing trend throughout the study.

### Histological analysis

The fish in G1 had increased HSMI heart scores from sampling IV (August) to sampling VII (November) (Table 1). Scoring of red muscle in G1 showed the same trend. Both heart and red muscle scores peaked in sampling VII. Both organs were below mildly affected (score 0.5–0.9) throughout the rest of the observation period.

**Table 1:** **Average histological scores from ten fish in each group, scored from 0–3 (none, mild, moderate, or severe) in heart and red muscle in G1 and G2 from sampling IV-XI (August 2020-May 2021)**

	Heart							
	*IV	V	VI	VII	VIII	IX	X	XI
**G1**	1	1	1.4	1.6	0.9	0.8	0.6	0.8
**G2**	1.5	0.1	0.2	0	0	0	0	0
	**Red muscle**							
	*IV	V	VI	VII	VIII	IX	X	XI
**G1**	0.2	0.9	0.75	1.4	0.5	0.9	0.5	0.5
**G2**	0.2	0.1	0.3	0.1	0.1	0.1	0	0

The fish in G2 had an elevated heart score in sampling IV (mild to moderate). Heart scores in the remaining samplings were low (<0.2). Only minor changes were seen in the red muscle of fish in G2 (<0.3). White muscle was generally without changes in both groups at all samplings, except for occasional single myocyte degeneration and occasional melano-macrophages in the endomysium.

### Blood parameters

**Figure 4:**
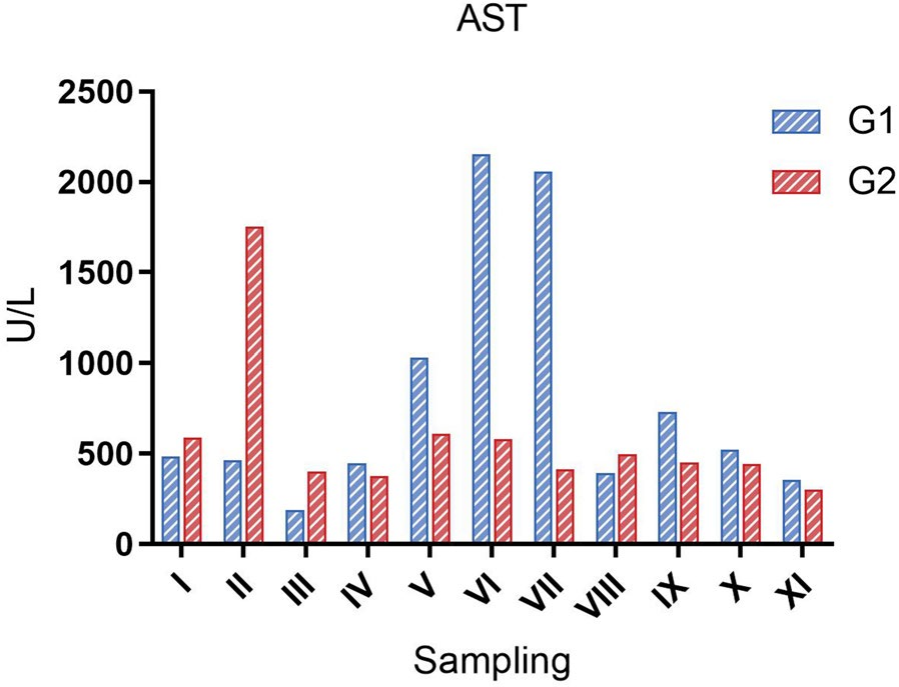
**AST levels in plasma at samplings I-XI.** Each bar represents absolute plasma levels (U/L) of a sample pooled from *n* = 10 fish.

The fish in G1 had increased levels of AST from sampling V (September) to sampling VII (November). The fish in G2 show elevated levels of AST at sampling II (June) but had lower levels from sampling III-XI (Figure 4). CK was also measured at all samplings, however, there were no elevated levels during the study (data not shown).

### Appetite, mortality, and clinical nutrition

**Figure 5:**
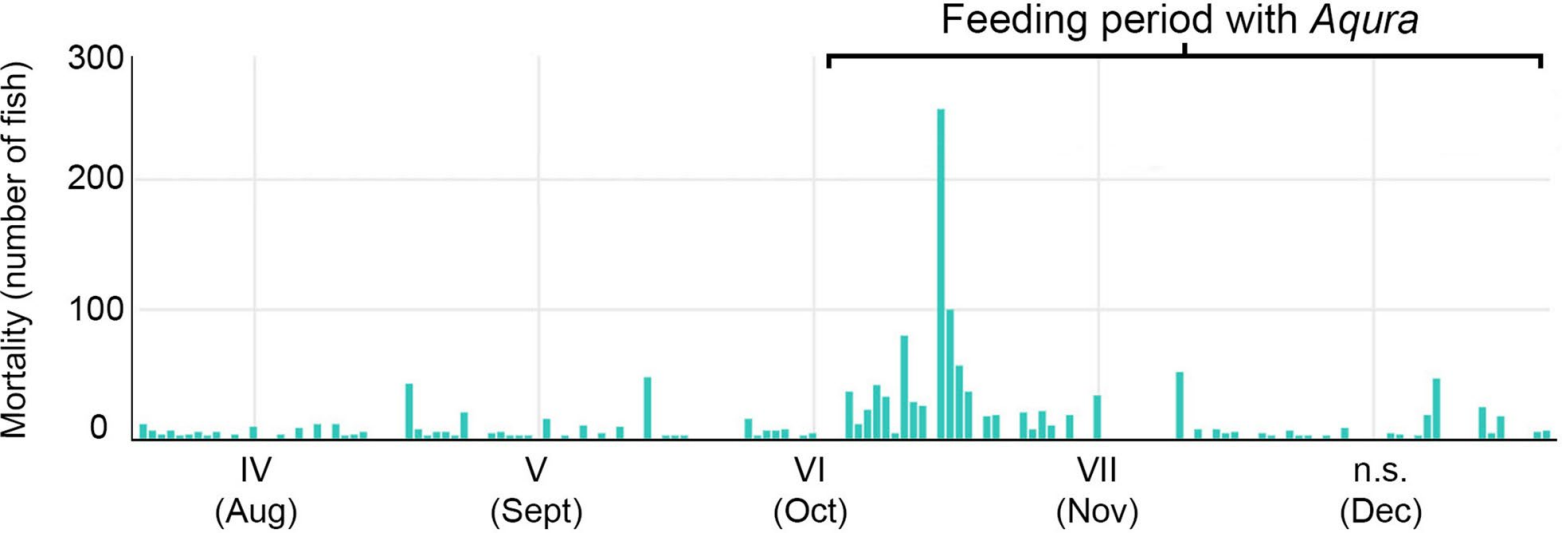
**Mortality registration in G1.** The feeding period with a clinical nutrition diet was from early October to mid-December. The outbreak of HSMI occurred during the same period, with increased mortality in October. Peak mortality was approximately 150 fish/day. The highest daily registration (~250 fish) is a result of accumulation of deceased fish from several days. n.s. = no sampling in December.

The appetite in G1 was normal during the first 5 weeks post sea transfer while a modest increase in mortality (3.77% accumulated mortality) was registered. The fish in G2 had decreased appetite and increased mortality (7.41% accumulated mortality) over the same period. From June to October, both apetite and mortality numbers reached normal production values and remained stable in G1 and G2. In October, an increase in mortality numbers was registered in G1 which was restricted to a very limited time-period (Figure 5). Clinical nutrition was applied in G1 in October, as the combined results from PRV-1 analysis, gene expression analysis, blood parameters and histology indicated the onset of HSMI. The difference in appetite between G1 and G2 from October to December during the outbreak of HSMI, i.e. during the clinical nutrition feeding period in G1, was negligible (data not shown).

## Discussion

The scope of this study was to forecast HSMI in farmed salmon and to apply clinical nutrition to counteract the impact of disease. An interdisciplinary fish health strategy was organized based on broad and frequent analysis of the fish health status, with daily monitoring of production parameters and monthly analysis of the population health. This included autopsy, testing of PRV-1 and immune gene expression status, histological analysis, and analysis of certain blood parameters related to skeletal muscle and heart disorders. We were able to predict the onset of an emerging HSMI outbreak which enabled us to implement a counter measure through clinical nutrition in an attempt to mitigate disease severity.

Populations of PRV-1 negative fish (G1) and positive fish (G2) were distributed to separate sea cages and were followed throughout the first 12 months at sea. The results show 100% detection of PRV-1 in fish in G1, 4 months post saltwater entry. A progressive increase in virus RNA load in G1, seen as a lowering trend of the Ct value of PRV-1, in addition to increased levels of the blood enzyme AST and increased expression of early anti-viral immune genes, was presumed to indicate an imminent HSMI outbreak. Importantly, clinical signs of disease at autopsy could not be observed at this time and production-related parameters such as appetite and mortality were normal. Subsequently, clinical nutrition was applied over a period of 10 weeks. The outbreak of HSMI was confirmed approximately 1 week into the clinical nutrition feeding period. The PRV-1 strain was sequenced, and the isolate was determined to be a high-virulence isolate, known to cause more severe histopathological changes than low-virulence isolates [10]. Sequence identities of PRV-1 isolates in G2 and G1, suggest the likely spread of virus from G2 to G1. Even though the isolate was classified as highly pathogenic, mortality remained low in G1, which could be due to the mitigating effect of clinical nutrition.

Throughout the study, the monitoring of fish health was organized to allow a proactive clinical approach. The monitoring differed from ordinary health surveillance, not only by monthly samplings and multiple laboratory analyses, but also by the structure of the fish health surveillance panel. The panel included on-site fish health personnel, veterinarians from the feed supplier company and veterinarians from the diagnostic laboratory. All results were obtained and combined within 1 week after each sampling and then analyzed and discussed by the fish health panel. This ensured a broad understanding at the time of the current fish health status, and a comprehensive decision basis with regards to the development of HSMI and when to apply clinical nutrition. Representative selections of fish were sampled, aiming to mirror the general health of the population, and not the recently diseased fish. This contrasts with common fish health practice focusing mainly on deceased fish, which is in line with fish health regulations (Regulations on the operation of aquaculture facilities § 13). The regulations state that a representative selection of moribund or recently deceased fish with aberrant behavior should be autopsied. Based on our results, we argue that an interdisciplinary fish health strategy with emphasis on different groups can predict the onset of HSMI, and thus can be beneficial for production and fish welfare.

PRV-1 infection and the development of HSMI were closely monitored in the G1 group. The fish in groups G2-G6 were regarded as the reservoir for PRV-1 on the farm, and G2 was treated equally to G1 in terms of samplings and methodology. In contrast to G1, G2 experienced increased mortality directly after sea transfer, but did not show clinical signs of disease throughout the rest of the study period. The increased mortality in G2 might have been due to an early outbreak of HSMI, though this could not be verified. PRV-1 Ct values and gene expression results from this period (sampling I-IV), in addition to increased histological heart scores in sampling IV (August), could very well agree with an HSMI diagnosis at an early stage. The PRV-1 isolate was genetically characterized as highly virulent, which further supports this hypothesis. On the contrary, HSMI is not commonly diagnosed immediately after sea transfer and may go unnoticed due to other common smolt-related problems. Løvoll et al. reported a prevalence of about 36% PRV-1 infection in selected freshwater cohorts [9]. Similarly, Patogen Analyse has reported a prevalence of 44–49% (unpublished data). In fact, in the Norwegian Fish Health Report from 2021 [1], PRV-1 infection was pinpointed as an increasing problem at certain smolt production sites struggling with HSMI. Thus, HSMI should not be neglected as a contributing cause to mortality in smolts.

The detection of PRV-1 in G1 went from 0 to 100% in 4 weeks (from sampling event III to IV), based on our sampling of ten fish. Sampling of ten fish is common practice when infectious diseases are monitored or suspected in the field; however, nuances in the prevalence of PRV-1 infection could have been detected if the sampling sizes were increased. In a similar field trial, Bjørgen et al. [8] found that the development of PRV-1 infection in PRV-1 naïve fish occurred over the course of 25 weeks. Here, the sampling size was ~ 60 fish per month. Importantly, the PRV-1 isolate was not sequenced in their study. In the present study, a highly virulent isolate was identified. The nucleotide sequence of the isolate in the present study was identical to that of a previously sequenced isolate (NOR2019 NRL-1742 m) originating in 2019 from the same geographical area as in the current study [11]. Identical isolates were found in G2 and G1, in line with horizontal spread of infection within the farm site. This raises some important questions regarding the current practice and fish health strategy. Early PRV-1 infection can potentially cause an equally early outbreak of HSMI, as might have been the case in G2 in this study. However, PRV-1 naïve fish are likely to develop HSMI at a later stage and thus also at a larger fish size, associated with larger economic losses. Hence, being in control of the PRV-1 status of different fish groups at the time of sea transfer seems crucial in the strategic fish health work and can be of aid to fish health practitioners in the handling of HSMI and other PRV-1 related conditions [20]. Other factors such as water currents, density of fish farms and health status of wild fish stocks etc. may also be important for the spread of PRV-1 but are far more difficult to control.

An imminent outbreak of HSMI in G1 was suspected based on increasing PRV-1 RNA loads, increased expression of antiviral immune genes and elevated plasma level of AST in sampling V (September). Based on these results, clinical nutrition was applied for 10 weeks. Importantly, mortality and appetite were assessed as normal and mild histopathological changes were detected at this time. As the mortality increased, histopathological scoring confirmed the HSMI diagnosis. This is in line with the development of HSMI in infection trials, where peak viral loads occur approximately 4–6 weeks prior to histopathological changes [21]. We found that genes associated with antiviral immune responses to RNA viruses, including *Mx*, *Gig2* and *Viperin*, were upregulated in the early phase of infection, followed by increased expression of *CD8α* and *GZMa* in the later stage. This was in line with previous studies on the dynamics of the innate and adaptive immune responses in PRV-1 infection [16, 17, 22, 23]. Cytotoxic T cells are believed to be involved in virus clearance, and increased expression of *CD8α* and *GZMa* has been found to correlate with decreasing PRV-1 RNA levels in the later stage of infection [23]. In addition to cellular immunity, clinical nutrition might have modulated the immune responses. The HSMI outbreak in G1 was of relatively short duration and the increased mortality was restricted to a limited period. Also, the histological scoring revealed that mild to moderate changes were most prevalent and that the changes gradually became less severe. The positive effects of increased EPA levels on HSMI-related changes have been reported previously in an HSMI infection trial [13], and likewise the positive effects of increased EPA and DHA in terms of growth performance, welfare, robustness, and overall quality [24]. The fish in G2 could also have benefitted from switching from a standard grower diet to clinical nutrition after sampling I, but this remains speculatory. Although pre-smolt and smolt diets in general have higher levels of EPA/DHA compared to grower diets, these levels are still below the minimum level of 14% EPA/DHA of total fat in the clinical nutrition diet used in our study. As this was a field study, a control group developing HSMI under identical conditions fed a standard grower diet was not available, and thus, all effects of clinical nutrition need to be interpreted with caution.

Trends in PRV-1 RNA and immune gene expression, histology scores and the level of blood enzymes were assessed from sampling to sampling. All these parameters contribute with information about the infection, but they differ as they provide key information at different timepoints. Importantly, knowing the progression of PRV-1 and particularly when peak viral infection occurred in G1, was crucial for forecasting the occurrence of HSMI and when to apply clinical nutrition. In fact, predicting the temporal nature of HSMI and the application of clinical nutrition can be based solely on monitoring PRV-1 levels in the population; however, additional information on increased anti-viral gene activity and increasing AST levels strengthened our suspicion. Also, sequencing of PRV-1 confirmed the presence of a high virulent strain in the population, with increased risk of a more severe manifestation of HSMI. Histological results and investigation of adaptive immune genes were only needed to confirm HSMI at a later stage. In sum, monitoring of all parameters were necessary to gain a holistic understanding of the course of the disease.

G1 and G2 were monitored closely for 12 months and HSMI was only confirmed (G1) or believed to occur (G2) at a single occasion in each group. To the best of our knowledge, no study has addressed whether HSMI can occur multiple times within a population. This is also debated among fish health personnel. Fish that undergo a PRV-1 infection will raise a specific immune response [25], however, the duration of the specific response is unknown, and the response is not sufficient to clear the infection [26, 27]. This can be explained by continuous viral transcription, but with arrest of viral translation, which has been shown in infection trials [28]. Based on our RT-qPCR results, similar mechanisms may apply during natural infections with PRV-1.

In conclusion, our results show that a proactive health surveillance, combining field data with laboratory-based methods such as blood-, gene- and histological analysis, makes it possible to stay in front of an imminent outbreak of HSMI and to apply measures to counteract the impact of disease at the right time. We argue that population-based health surveillance in aquaculture should aim to be prospective, which is underlined by our successful approach in dealing with HSMI.

## Supplementary Information

**Additional file 1. Sequences of primers used in quantitative RT-PCR analysis.**

## Data Availability

Excess data beyond the results presented in the paper is available upon request to corresponding author.
