# Supplementary material for: In-depth health surveillance and clinical nutrition in farmed Atlantic salmon: a strategic attempt to detect and mitigate an HSMI outbreak
Source: Vet Res. 2023 Jan 24;54:3. doi: 10.1186/s13567-023-01137-1 (PMC9872415; doi:10.1186/s13567-023-01137-1)
Supplement: Supplementary file 1 — Additional file 1. Sequences of primers used in quantitative RT-PCR analysis. [file 13567_2023_1137_MOESM1_ESM.docx]

| **Name** | **Accesion number** | **Forward primer** | **Reverse primer** |
| --- | --- | --- | --- |
| Viperin (rsad2) | XM_045723372.1 | AGCAATGGCAGCATGATCAG | TGGTTGGTGTCCTCGTCAAAG |
| Gig2 | XM_014209594.2 | GATGTTTCATGGCTGCTCAA | CTTTTCGGATGTCCCGACTA |
| MX1-2 | U66475; U66476 | GATGCTGCACCTCAAGTCCTATTA | CGGATCACCATGGGAATCTGA |
| CD8β | AY693394 | CCAAAAGGAACGATCAAACCC | TGTAGATCAAAGCCGCAGCC |
| GZMa | NM_001141037.1 | GGCGGTGAAACCTCTAGCGT | GCATTGTTCTTGGTGGCTCC |
| ETIF3 | NM_001141695.1 | ACCCAGAGACCACCAAACAG | GCCCCAGAGTAGTTTCCACA |
| Actin | XM_014194536.2 | CAACTGGGACGACATGGAGA | AGTGAGCAGGACTGGGTGCT |
